# Down Syndrome Births Among Live Births from the CDC Wonder Database

**DOI:** 10.3390/children13050612

**Published:** 2026-04-28

**Authors:** Stephanie L. Santoro, Chance Alvarado, Stephen A. Hart, Thomas Casto, Clifford L. Cua

**Affiliations:** 1 Massachusetts General Hospital, Boston, MA 02114, USA; ssantoro3@mgh.harvard.edu; 2 Department of Pediatrics, Harvard Medical School, Boston, MA 02115, USA; 3 Nationwide Children’s Hospital Heart Center, Nationwide Children’s Hospital, Columbus, OH 43205, USA; chance.alvarado@osumc.edu (C.A.); stephen.hart@nationwidechildrens.org (S.A.H.); thomas.casto@nationwidechildrens.org (T.C.); 4 Center for Biostatistics, The Ohio State University Wexner Medical Center, Columbus, OH 43210, USA; 5 Biostatistics Resource at Nationwide Children’s Hospital, Abigail Wexner Research Institute, Nationwide Children’s Hospital, Columbus, OH 43205, USA

**Keywords:** Down syndrome, birth rate

## Abstract

We evaluated the birth rate of Down syndrome (DS) in the CDC birth certificate online database. From 2016 to 2025, live birth incidence could range greatly (depending on the proportion of unknown cases that are counted as DS+) due to relatively high numbers of unknown/not stated status. The annual live birth incidence of DS in live-born infants using CDC birth certificate data from 2016 to 2025 shows a wide range of potential birth rates as calculated here, due to relatively high numbers of unknown/not stated DS status. Although our findings overlap with published data, future studies are needed to further evaluate the current birth rate of DS in the US.

## 1. Introduction

Down syndrome (DS) is the most common and viable human trisomy, with an incidence of approximately 1 per 700 live births in the United States [1]. Patients with DS have an increased risk of anatomical as well as physiologic abnormalities compared to the general population, which places them at increased risk of morbidity and mortality [2,3,4,5,6,7,8,9,10,11,12,13,14,15,16].

The DS birth rate has been studied through various methodological approaches. Since 1979, data from population-based birth defects surveillance programs, most recently titled the National Birth Defects Prevention Network (NBDPN), have existed [17,18,19,20,21,22].Over time, the NBDPN has had varying numbers of states/regions included and varying types of surveillance (active or passive) by location. In its most recent published study (2016–2020), the NBDPN included 13 US population-based birth defects programs with active or a combination of active and passive case ascertainment methods that included data on all birth outcomes [22]. Additionally, Besser et al. described findings from 1979 to 2003 from one city, the Metropolitan Atlanta Congenital Defects Program (MACDP) [23]. A third methodology involved data modeling from 1900 to 2010 using “Data on the total live births in the U.S. from 1909 onwards from the U.S. Census Bureau, Vital Statistics of the United States, Birth Data Files, National Center for Health Statistics, CDC” to model live birth data in 2006–2010 [24].

The DS live birth rate from those published studies to date has ranged from: 8.3 per 10,000 births (1 in 1204 in 2003) [23], 10.3 per 10,000 births (1 in 971 from 1979 to 2003) [21], to 14.47 per 10,000 births (1 in 691 from 2004 to 2006) [20]. The use of existing datasets and modeling to predict future live birth rates in the United States from 1900 to 2010 found the live birth prevalence for DS in the most recent years (2006–2010) to be 12.6 per 10,000 births (95% CI 12.4–12.8; equivalent to 1 in 794), with around 5300 births and 3100 DS-related elective pregnancy terminations annually [24].

The goal of this study was to determine the annual birth rate of infants with DS in the United States using a data source that has not been used before, nor have findings from this dataset published to date, and to summarize past birth rate research, comparing our findings to other published sources for historical context. As we describe in our methodology, the CDC Wonder database provides a unique perspective on live birth data, which differs from past registry-based surveillance studies but may also have limitations, such as the potential for misclassification.

## 2. Methods

Information related to live births was obtained from the publicly available Natality Information–Live Births databases hosted by Centers for Disease Control and Prevention (CDC) Wonder [25,26]. These databases are sourced from birth certificate data and consist of live births occurring within the 50 U.S. states and D.C to U.S. residents. Data source: Centers for Disease Control and Prevention, National Center for Health Statistics. National Vital Statistics System, Natality on CDC Wonder Online Database. Data are from the Natality Records for births occurring in 2023 through last month, as compiled from data provided by the 57 vital statistics jurisdictions through the Vital Statistics Cooperative Program. In the CDC Wonder dataset, individuals can be classified with a diagnosis of DS on birth certificates as confirmed, pending, unknown/not stated, or no. DS diagnosis was available in the CDC Wonder dataset beginning in 2016.

In August 2025, our team retrieved finalized data from 2016 to 2022 and provisional data from 2023 to May 2025 for all recorded births. Provisional births are based on the flow of data into the system; it can take several days for birth records to be submitted to the National Center for Health Statistics (NCHS), processed, edited, and tabulated. Provisional data may be incomplete, but birth counts for earlier months are continually revised as new and updated birth certificate data are received from the states by NCHS.

### Analysis

Counts of total live births and counts by DS diagnosis type (confirmed, pending, unknown/not stated, or no) from CDC Wonder were stratified by year. The annual live birth incidence of DS was calculated by dividing the number of individuals with DS (i.e., DS+) by the number of total births. Based on the information available from CDC Wonder, this was calculated in three ways: (1) the minimum birth rate with only those with confirmed DS counted as DS+; (2) a presumptive birth rate with those with confirmed or pending DS counted as DS+; and (3) the maximum birth rate with those with confirmed, pending or unknown DS categorization counted as DS+. Then, three versions of calculation (3) were conducted with varying fractions of those categorized as unknown included: ¼, ½, and ¾ of unknown diagnoses were counted as DS+. This was performed to elucidate the potential magnitude of bias presented given instances of live births being classified as unknown or not stated as opposed to confirmed, pending or no DS

The Institutional Review Board determined that this research was not considered human research, and approval was not required. Datasets were obtained as public-use data files and accessed on 18 June 2025, and then re-retrieved on 25 August 2025, to maximize the provisional data for 2025 available for analysis.

## 3. Results

Among the over 35 million live births in the U.S. from 2016 to 2025 in the CDC Wonder database, there were 8363 confirmed DS births (0.02%), 10,257 pending DS births (0.03%), and 67,298 unknown or not stated DS births (0.2%), yielding a range of birth rates from a maximum possible rate of 1 in 410 (if counting all unknown cases as DS+) to a minimum possible rate of 1 in 4217 (if only counting those with confirmed DS as DS+; Table 1). 

**Figure 1:**
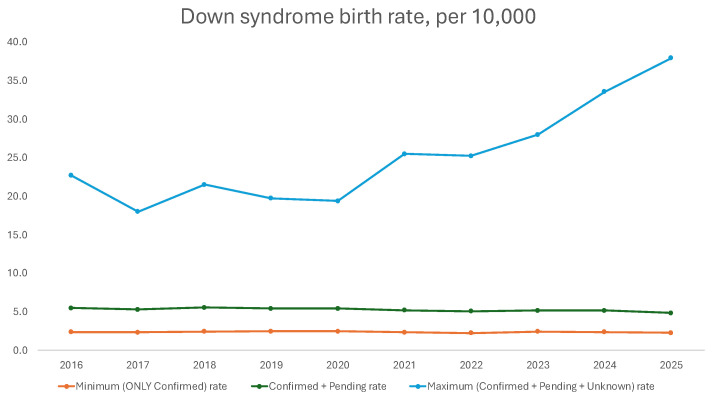
Down syndrome birth rate in 2016 to 2025 from the CDC Wonder database, calculated in three ways: (1) the minimum rate including only those with confirmed DS, (2) the presumptive rate including those with confirmed and pending DS status, and (3) the maximum rate including those with confirmed, pending or unknown DS status.

**Figure 2:**
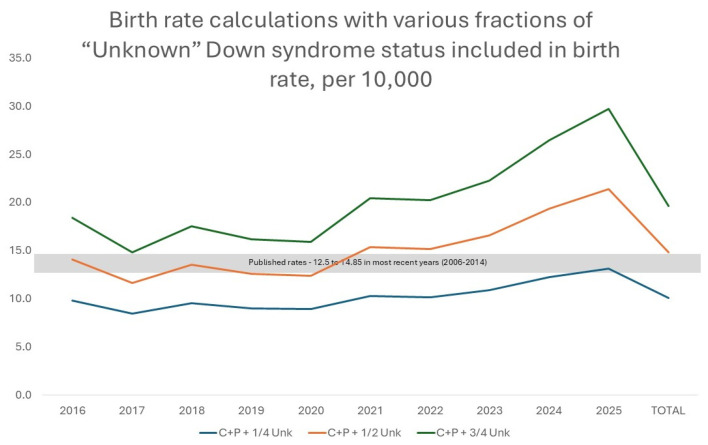
Down syndrome birth rates calculated with varying fractions (¼, ½ or ¾) of “unknown” DS status included in the birth rate calculation, along with confirmed (C) and pending (P) DS status. Data from the CDC Wonder database from 2016 to 2025.

Minimum DS birth rates per year ranged from 2.2 to 2.5 per 10,000 live births, while the presumptive DS birth rates per year ranged from 4.8 to 5.6 per 10,000 live births (Table 1); both were generally stable over the time period studied (Figure 1). The maximum DS birth rates per year ranged from 18.0 in 2017 to 37.9 in 2025, acknowledging that more recent 2025 data could be impacted by provisional data (Table 1). The maximum DS birth rate showed an increasing trend over time (Figure 1). Varying the fraction (¼, ½, or ¾) of those categorized as unknown was graphed (Figure 2). From 2016 to 2022, the live birth incidence of DS for minimum, presumptive, and maximum rates was 2.4, 5.3, and 21.7 cases per 10,000 live births, respectively, which is comparable to the total rates derived including contemporary and provisional data.

We summarized data from the published literature on DS birth rates (Table 2); in the recent literature using data from a population-based birth defects surveillance program (2006–2020) and dataset modeling (2006–2010), the published DS birth rate was 12.5–15.5 per 10,000 births, which is equivalent to 1 in 645 to 1 in 800.

Graphing published DS birth rate data and our findings from the CDC Wonder dataset, published birth rate data fall within the range of DS birth rates (between the presumptive DS birth rate and the maximum DS birth rate) from our calculations (Supplemental Figure S1).

## 4. Discussion

We began this study because past publications have used population-based contact registries in some states and regions to passionately follow birth rate data at locations around the US, but none to-date have had data from all 50 states. Other modeling studies from 2010 or before have projected birth rate data based on several data sources, but the DS birth rate from the most direct source, the CDC Wonder dataset of birth certificate data, has not been previously described in the medical literature.

Through our analysis of CDC Wonder data from 2016 to May 2025, we found that DS occurred in 1 of 1895 infants (5.3 per 10,000 births), presuming that those with a pending DS status would likely be confirmed to have DS once pending testing was returned to clinicians. The minimum and presumptive DS birth rates remained stable during the time period of this study. However, if we calculated the DS birth rate including those with unknown DS diagnosis, the maximum birth rate in our cohort for the total time period was 24.4 per 10,000 live births. In our calculations, the maximum DS birth rate relies on the assumption that all or nearly all infants with unknown DS status truly have DS. While this may not be an epidemiologically plausible rate, this upper bound quantifies the uncertainty associated with the categorization of unknown DS.

Past studies have found a DS birth rate ranging from 12.5 to 14.85 per 10,000 births in recent years. For our data to align with published rates, approximately half of unknown DS status would mathematically need to truly have DS. Given that DS is a relatively well-known genetic syndrome and has very recognizable facial features, we would not suspect a practicing clinician to label an infant with DS as unknown, though this is theoretically possible if half of the 67k infants (i.e., 33k infants) with DS were incorrectly categorized as unknown status on their birth certificates. In this instance, future studies could analyze these unknown cases to guide any needed interventions. For example, if clinical uncertainty (i.e., waiting for confirmatory molecular results rather than giving a diagnosis based on physical exam) is a common source of reporting unknown status, then additional clinician education on clinical features of DS and earlier diagnosis by clinical features alone may be helpful. It is also possible that other causes of unknown documentation are more common, such as administrative coding issues, delayed cytogenetic confirmation or variation in reporting practices between states. Regardless, it is important to identify and document the diagnosis of DS at the time of birth (i.e., add to their birth certificate diagnoses), to avoid delays in families connecting to perinatal support groups, genetic counseling, and resources that are legally mandated in many states [28] and known to improve families’ experiences [29].

Additionally, at the time of this project, the most recent published DS birth rates using direct data were from 2020 or earlier, without current data from the most recent years. Continuing to track and follow DS birth rates over time is especially relevant given the findings from Stallings et al., which summarized recent national prevalence per 10,000 live births (and 95% CI) during four time periods: 12.78 (12.34–13.22) from 1999 to 2001, 13.56 (13.20–13.92) from 2004 to 2006, 14.14 (13.81–14.48) from 2010 to 2014, and 15.55 (15.37–15.73) from 2016 to 2020 [22]. Their data suggest that the DS birth rate is increasing and that DS is becoming more prevalent over the last two decades. However, the CDC Wonder dataset we analyzed does not show substantial changes in the minimum or presumptive DS birth rate over time.

Our study is limited by the type of data collected and recorded in the CDC Wonder database, relying on birth certificate data, though the CDC Wonder database has been used in other published research [29,30,31,32]. Although the data in CDC Wonder are de-identified without detailed demographics, we are hopeful that future studies could take more variables into account, such as maternal age and geography. We demonstrate the use of the CDC Wonder dataset to estimate live birth incidence of DS compared to other registries in the US. Birth certificate data are a snapshot in time and have been shown to have limitations but also to be a useful tool to study DS [33,34]. Our methodology (CDC Wonder birth certificate data) differs from that of population-based congenital anomaly registries, which have inherent differences that we hypothesize could lead to differences in study outcomes (e.g., the ability to contact those in a registry would allow confirmation or exclusion of unknown cases, the information collected and level of detail available, the source of the information, and the way the population is captured). To better understand how our findings using birth certificate data compare/contrast to past studies using birth registries, future work may be useful to evaluate the use of both approaches, as there may be times when one or the other is preferred.

With the potential for birth certificates to be misclassified, leading to underreporting of DS on birth certificates, it would be ideal to follow those with pending or unknown DS status prospectively to determine how often those individuals go on to be diagnosed with DS, as, in our experience, birth certificates are typically completed within 24–72 h after birth. Future studies could explore the DS birth rate through other means to validate or support our findings, which show a birth rate (5.3 infants per 10,000 live births, or 1 in 1895), while the birth rate generally cited in go-to references for DS is approximately 1 in 800 live births [2].

## 5. Conclusions

The annual live birth incidence of DS in live-born infants using CDC birth certificate data from 2016 to 2025 shows a wide range of potential birth rates, as calculated here, due to relatively high numbers of unknown/not stated DS status. Although our findings overlap with published data, future studies are needed to further evaluate the current birth rate of DS in the US.

## Figures and Tables

**Table 1:** Down Syndrome Status for Births form All States in CDC Database.

	Down Syndrome Status on Birth Certificate					Minimum DS Birth Rate (C Only)/T		Presumptive DS Birth Rate (C + P)/T		Maximum DS Birth Rate (C + P + U)/T	
Year	Confirmed	Pending	Unknown or Not Stated	No	Total Births	X in 10,000	1 in X	X in 10,000	1 in X	X in 10,000	1 in X
2016	937	1227	6791	3,936,920	3,945,875	2.4	4211	5.5	1823	22.7	441
2017	898	1141	4897	3,848,564	3,855,500	2.3	4293	5.3	1891	18.0	556
2018	921	1185	6044	3,783,562	3,791,712	2.4	4117	5.6	1800	21.5	465
2019	920	1104	5364	3,740,152	3,747,540	2.5	4073	5.4	1852	19.7	507
2020	894	1060	5048	3,606,645	3,613,647	2.5	4042	5.4	1849	19.4	516
2021	856	1045	7439	3,654,952	3,664,292	2.3	4281	5.2	1928	25.5	392
2022	818	1039	7399	3,658,502	3,667,758	2.2	4484	5.1	1975	25.2	396
2023	867	988	8112	3,586,037	3,596,004	2.4	4148	5.2	1939	28.0	357
2024	853	1016	10,268	3,606,130	3,618,267	2.4	4242	5.2	1936	33.5	298
2025 *	399	452	5837	1,755,696	1,762,384	2.3	4417	4.8	2071	37.9	264
TOTAL	8363	10,257	67,298	35,177,074	35,262,992	2.4	4217	5.3	1894	24.4	410

* Only January to July available for 2025.

**Table 2:** Summary of Past Studies Describing Live Birth Rates of Down Syndrome.

First Author	Years Studied	Methodology	Data Source	Location Studied	Birth Rate of DS—per 10,000 Live Births	1 in X
Shin [21]	1979–2003	Retrospective review	Data from population-based birth defects registries	10 programs in 10 states/regions	11.8 per 10,000 live births in 1999–2003 1979: 9.0 at birth 2003: 11.8 at birth Overall prevalence: 10.3 per 10,000 age 0–19 yrs	971
CDC MMWR [27]	1983–1990	Retrospective review	Data from population-based birth defects surveillance programs	17 states	9.2 cases per 10,000 live-born infants Variation among states (range: 5.9 [Kansas] to 12.3 [Colorado]). Rates differed significantly by racial/ethnic group (*p* < 0.001, Chi-square test): for Hispanic infants, the rate of DS was 11.8; for white infants, 9.2, and for black infants, 7.3.	1087
Canfield [17]	1999–2001	Retrospective review of data with adjustments based on US live birth population	National Birth Defects Prevention Network data	35 US surveillance programs, data from 22 states; adjusted data from 11 states	13.65 per 10,000 live births 11.82 using passive without follow-up 12.94 using active case-finding 12.78 adjusted for race and ethnicity 13.65 adjusted for maternal age	733
Besser [23]	1979–2003	Retrospective review	Population-based birth defects registry	Atlanta: Metropolitan Atlanta Congenital Defects Program (MACDP)	8.3 per 10,000 2003: 13.0 at birth 8.9 in 1979–1983 11.6 in 1999–2003	1204
Parker [20]	2004–2006	Retrospective review of data with adjustments based on US live birth population	National Birth Defects Prevention Network data	24 US surveillance programs in 24 states; adjusted data from 14 programs	14.47 per 10,000 live births 13.08 with passive case-finding 13.48 using active case-finding 13.56 adjusted for race and ethnicity 14.47 adjusted for maternal age 13.51 per live births from 3 states	691
Mai [19]	2006–2010		National Birth Defects Prevention Network(NBDPN) Congenital Malformations Surveillance Report	41 programs	12.5 per 10,000 live births 12.17 of live births from 2000–2004 (6 states) 12.33 of live births from 2006–2010 (6 states) 14.2 per 10,000 for all pregnancy outcomes	800
De Graaf [24]	2006–2010	Dataset modeling of data from 1900–2010	Data on the total live births in the U.S. from 1909 onwards from the U.S. Census Bureau, Vital Statistics of the United States, Birth Data Files, National Center for Health Statistics, CDC used in modeling	US	Estimates and model predictions: 12.6 per 10,000 (2006–2010) As of 2007, our model would predict 19.1 per 10,000 for live births	794
Mai [18]	2010–2014	Retrospective review of data with adjustments based on US live birth population	National Birth Defects Prevention Network (NBDPN)	39 US surveillance programs	14.85 per 10,000 live births 14.14 live births when adjusted for maternal race/ethnicity 15.74 live births when adjusted for maternal age	673
Stallings [22]	2016–2020	Retrospective review of data with adjustments based on US live birth population	National Birth Defects Prevention Network (NBDPN)	13 US population-based birth defects programs with active or a combination of active and passive case ascertainment methods that included data on all birth outcomes	15.55 cases per 10,000 live births All race/ethnicity and all ages: 16.33 (15.94–16.72 CI) 15.55 adjusted for maternal race/ethnicity 17.19 when adjusted for maternal age They “prevalence changes over four distinct birth cohort periods” and conclude that “The current analysis demonstrated continued increases in AVSD and trisomy 21” They note that “Caution should be used when directly comparing the three previous cohorts to the current cohort. Methodology was generally similar between this paper and the previous paper; however, this analysis used stricter criteria for the inclusion of programs when considering pregnancy outcomes, which could affect the observed prevalence”	643

Light gray indicate studies using continuations of an ongoing population-based registry; Gray indicates a population-based registry from one city (Atlanta); Blue indicates a study using data modeling from various sources of existing data.

## Data Availability

All utilized project data were retrieved from publicly available datasets.

## Supplementary Materials

The following supporting information can be downloaded at: https://www.mdpi.com/article/10.3390/children13050612/s1, Figure S1: Down syndrome birth rates from published literature and this study.

## References

[1] de Graaf G., Buckley F., Skotko B.G. (2017). Estimation of the number of people with Down syndrome in the United States. Genet. Med..

[2] Bull M.J. (2020). Down Syndrome. N. Engl. J. Med..

[3] Ferencz C., Rubin J.D., McCarter R.J., Brenner J.I., Neill C.A., Perry L.W., Hepner S.I., Downing J.W. (1985). Congenital heart disease: Prevalence at livebirth: The Baltimore-Washington Infant Study. Am. J. Epidemiol..

[4] Messick E.A., Hart S.A., Strominger J., Conroy S., Backes C.H., Cua C.L. (2025). Morbidity and Mortality in Neonates with Down Syndrome vs Those Without Down Syndrome by Gestational Age. Pediatr. Open Sci..

[5] Messick E.A., Hart S.A., Strominger J., Conroy S., Backes C.H., Cua C.L. (2025). Gestational age-based outcomes of neonates with Down syndrome in the neonatal intensive care unit (NICU): Review of pediatric health information system (PHIS) database. J. Perinatol..

[6] Rubin J.D., Ferencz C., McCarter R.J., Wilson P.D., Boughman J.A., Brenner J.I., Neill C.A., Perry L.W., Hepner S.I., Downing J.W. (1985). Congenital cardiovascular malformations in the Baltimore-Washington area. Md. Med. J..

[7] Swigonski N.L., Kuhlenschmidt H.L., Bull M.J., Corkins M.R., Downs S.M. (2006). Screening for celiac disease in asymptomatic children with Down syndrome: Cost-effectiveness of preventing lymphoma. Pediatrics.

[8] Weijerman M.E., van Furth A.M., van der Mooren M.D., van Weissenbruch M.M., Rammeloo L., Broers C.J., Gemke R.J. (2010). Prevalence of congenital heart defects and persistent pulmonary hypertension of the neonate with Down syndrome. Eur. J. Pediatr..

[9] Weijerman M.E., van Furth A.M., Vonk Noordegraaf A., van Wouwe J.P., Broers C.J., Gemke R.J. (2008). Prevalence, neonatal characteristics, and first-year mortality of Down syndrome: A national study. J. Pediatr..

[10] Bull M.J., Committee on Genetics (2011). Health supervision for children with Down syndrome. Pediatrics.

[11] Mekonnen T., Kristjansson D., Bratsberg B., Kohler H.P., Selbaek G., Larsen F.K., Langballe E.M., Engeland J., Kirkevold O., Haberg A.K. (2025). Changes in survival probabilities and mortality risks among population living with Down syndrome born 1967-2018: A Norwegian registry-based study. BMC Public Health.

[12] Tsou P.Y., Wang Y.H., Tapia I.E. (2025). Inpatient outcomes among children with Down syndrome: A Kids’ Inpatient Database study. BMC Pediatr..

[13] Ye E., Wu E., Han R. (2025). Global, regional, and national impact of Down syndrome on child and adolescent mortality from 1980 to 2021, with projections to 2050: A cross-sectional study. Front Public Health.

[14] Tantalean Da Fieno J., Leon Paredes R., Palomo Luck P., Del Aguila Villar C., Rizo Patron E. (2024). Down syndrome and outcomes in critically ill pediatric patients. Front. Pediatr..

[15] Gaechter P., Ebrahimi F., Kutz A., Szinnai G. (2025). Hospitalizations in people with down syndrome across age groups: A population-based cohort study in Switzerland. eClinicalMedicine.

[16] Goldberg S.W., Chalak C., Anderson B.R., Elhoff J., Gaydos S., Lubert A.M., Sassalos P., Gauvreau K., Gurvitz M. (2025). Outcomes in Adult Congenital Heart Disease Patients with Down Syndrome Undergoing a Cardiac Surgical Procedure. Ann. Thorac. Surg..

[17] Canfield M.A., Honein M.A., Yuskiv N., Xing J., Mai C.T., Collins J.S., Devine O., Petrini J., Ramadhani T.A., Hobbs C.A. (2006). National estimates and race/ethnic-specific variation of selected birth defects in the United States, 1999-2001. Birth Defects Res. Part A Clin. Mol. Teratol..

[18] Mai C.T., Isenburg J.L., Canfield M.A., Meyer R.E., Correa A., Alverson C.J., Lupo P.J., Riehle-Colarusso T., Cho S.J., Aggarwal D. (2019). National population-based estimates for major birth defects, 2010–2014. Birth Defects Res..

[19] Mai C.T., Kucik J.E., Isenburg J., Feldkamp M.L., Marengo L.K., Bugenske E.M., Thorpe P.G., Jackson J.M., Correa A., Rickard R. (2013). Selected birth defects data from population-based birth defects surveillance programs in the United States, 2006 to 2010: Featuring trisomy conditions. Birth Defects Res. Part A Clin. Mol. Teratol..

[20] Parker S.E., Mai C.T., Canfield M.A., Rickard R., Wang Y., Meyer R.E., Anderson P., Mason C.A., Collins J.S., Kirby R.S. (2010). Updated National Birth Prevalence estimates for selected birth defects in the United States, 2004-2006. Birth Defects Res. Part A Clin. Mol. Teratol..

[21] Shin M., Besser L.M., Kucik J.E., Lu C., Siffel C., Correa A., Congenital Anomaly Multistate P., Survival C. (2009). Prevalence of Down syndrome among children and adolescents in 10 regions of the United States. Pediatrics.

[22] Stallings E.B., Isenburg J.L., Rutkowski R.E., Kirby R.S., Nembhard W.N., Sandidge T., Villavicencio S., Nguyen H.H., McMahon D.M., Nestoridi E. (2024). National population-based estimates for major birth defects, 2016-2020. Birth Defects Res..

[23] Besser L.M., Shin M., Kucik J.E., Correa A. (2007). Prevalence of down syndrome among children and adolescents in metropolitan Atlanta. Birth Defects Res. Part A Clin. Mol. Teratol..

[24] de Graaf G., Buckley F., Skotko B.G. (2015). Estimates of the live births, natural losses, and elective terminations with Down syndrome in the United States. Am. J. Med. Genet. Part A.

[25] Centers for Disease Control and Prevention, National Center for Health Statistics (2022). National Vital Statistics System, Natality on CDC WONDER Online Database. Data are from the Natality Records 2016–2022, as compiled from data provided by the 57 vital statistics jurisdictions through the Vital Statistics Cooperative Program. https://wonder.cdc.gov/natality-expanded-current.html.

[26] Centers for Disease Control and Prevention, National Center for Health Statistics (2024). National Vital Statistics System, Provisional Natality on CDC WONDER Online Database. Data are from the Natality Records for births occurring in 2023 through last month, as compiled from data provided by the 57 vital statistics jurisdictions through the Vital Statistics Cooperative Program. https://wonder.cdc.gov/natality-expanded-provisional.html.

[27] Centers for Disease Control and Prevention (CDC) (1994). Down syndrome prevalence at birth—United States, 1983–1990. MMWR Morb. Mortal. Wkly. Rep..

[28] Lehman A., Leach M., Santoro S.L. (2021). Delivering a new diagnosis of Down syndrome: Parent experience. Am. J. Med. Genet. A.

[29] Gamber R.A., Blonsky H., McDowell M., Lakshminrusimha S. (2024). Declining birth rates, increasing maternal age and neonatal intensive care unit admissions. J. Perinatol..

[30] Okobi O.E., Ibanga I.U., Egbujo U.C., Egbuchua T.O., Oranu K.P., Oranika U.S. (2023). Trends and Factors Associated with Mortality Rates of Leading Causes of Infant Death: A CDC Wide-Ranging Online Data for Epidemiologic Research (CDC WONDER) Database Analysis. Cureus.

[31] Tseng S.Y., Alvarado C., Conroy S., Kistler I., Hart S.A., Fichtner S., Cua C.L. (2025). The Dobbs Decision and Incidence of Live Births with Cyanotic Congenital Heart Disease. Pediatr. Open Sci..

[32] Santoro S.L., Alvarado C., Tseng S.Y., Conroy S., Kistler I., Hart S.A., Fichtner S., Cua C.L. (2026). Down syndrome birth rate post Dobbs decision: Has it changed?. J. Perinatol..

[33] Ammar L., Bird K., Nian H., Maxwell-Horn A., Lee R., Ding T., Riddell C., Gebretsadik T., Snyder B., Hartert T. (2024). Development and Validation of a Diagnostic Algorithm for Down Syndrome Using Birth Certificate and International Classification of Diseases Codes. Children.

[34] Watkins M.L., Edmonds L., McClearn A., Mullins L., Mulinare J., Khoury M. (1996). The surveillance of birth defects: The usefulness of the revised US standard birth certificate. Am. J. Public Health.

