# Supplementary figures and images for: Down Syndrome Births Among Live Births from the CDC Wonder Database
Source: Children (Basel). 2026 Apr 28;13(5):612. doi: 10.3390/children13050612 (PMC13204170; doi:10.3390/children13050612)

Supplemental Figure      Down Syndrome Birth Rate, per 10,000

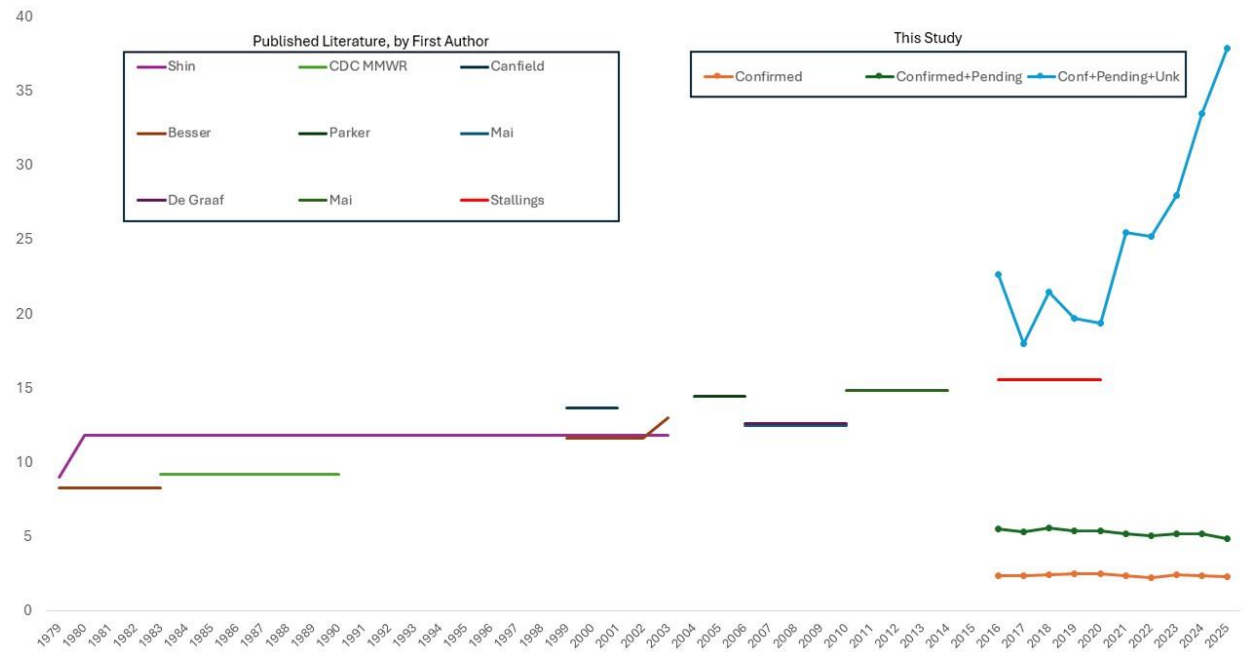

Supplement: Supplementary file 1 [file children-13-00612-s001.zip › children-4188094-supplementary.pdf]
